# TGF-β/BAMBI pathway dysfunction contributes to peripheral Th17/Treg imbalance in chronic obstructive pulmonary disease

**DOI:** 10.1038/srep31911

**Published:** 2016-08-23

**Authors:** Jian-Chu Zhang, Gang Chen, Long Chen, Zhao-Ji Meng, Xian-Zhi Xiong, Hong-Ju Liu, Yang Jin, Xiao-Nan Tao, Jiang-Hua Wu, Sheng-Wen Sun

**Affiliations:** 1 Department of Respiratory Medicine, Union Hospital, Tongji Medical College, Huazhong University of Science and Technology, 1277 JieFang Avenue, Wuhan 430022, China

## Abstract

BMP and activin membrane-bound inhibitor (BAMBI) is postulated to inhibit or modulate transforming growth factor β (TGF-β) signaling. Furthermore, strong upregulation of BAMBI expression following *in vitro* infection of chronic obstructive pulmonary disease (COPD) lung tissue has been demonstrated. In this study, we investigated whether TGF-β/BAMBI pathway is associated with COPD. Blood samples were obtained from 27 healthy controls (HC), 24 healthy smokers (HS) and 29 COPD patients. Elevated Th17/Treg ratios, and increased levels of BAMBI protein and mRNA (in plasma and CD4^+^ T cells respectively), were observed in COPD compared with HC and HS. BAMBI expression was first observed on human CD4^+^ T cells, with a typical membrane-bound pattern. The enhanced plasma BAMBI levels in COPD positively correlated with the increased plasma TGF-β1 levels and Th17/Treg ratio. Together, an impaired TGF-β/BAMBI pathway may promote the inflammation leading to Th17/Treg imbalance, which is a new mechanism in smokers who develop COPD.

Chronic obstructive pulmonary disease (COPD) is currently the fourth leading cause of mortality worldwide but is projected to become the third leading cause by 2020^1^. Although the role of non-specific innate immune processes mediated by neutrophils and macrophages in COPD has been well established^2^, susceptibility to COPD has been hypothesized to develop by a transformation from the innate immune responses present in smokers toward an adaptive immune response with features typical of autoimmune inflammation^3^. Paradoxically, not all human smokers develop emphysema, which is in contrast to a mouse model indicating that smoke exposure in mice is sufficient to induce lung disease^4^. Recently, certain antigens induced by tobacco smoke or infection have been suggested to be responsible for T lymphocyte activation^5^,^6^; however, the precise regulation of inflammation in COPD pathogenesis remains controversial.

In addition to T helper (Th) 1 and Th2 cells, which are involved in immunopathologic inflammation^7^, extensive evidence has suggested critical roles for Th17 and regulatory T cell (Treg) subsets in immune system regulation^8^,^9^,^10^. Previous studies have reported different numbers of Treg and Th17 cells in lung tissue, bronchoalveolar lavage (BAL), or peripheral blood in COPD^11^,^12^,^13^,^14^,^15^. Meanwhile, increased Th17 cell levels have been inversely correlated with reduced Treg levels in COPD^16^,^17^, suggesting that the imbalance between these two subsets might contribute to COPD pathogenesis. Despite these accumulating data, specific factors affecting Th17/Treg imbalance in COPD have attained a further level of complexity.

As a multifunctional cytokine that is involved in a variety of human diseases^18^, transforming growth factor β (TGF-β) plays a pivotal role in the differentiation of naive CD4^+^ T cells into Treg or Th17 cells, which is indeed dependent on the inflammatory microenvironment and on epigenetic modifications^19^. Elevated TGF-β1 expression has been observed in small airway epithelium, lung tissue and peripheral blood from COPD patients compared with healthy smokers or nonsmokers^20^,^21^,^22^,^23^; however, this was not observed in several studies^24^,^25^. In particular, the lung tissues of COPD patients display stronger expression levels of BMP and activin membrane-bound inhibitor (BAMBI)^26^, which is a 260-amino acid transmembrane protein that acts as a competitive receptor antagonist for TGF-β type-I receptors (TGF-β RI) and the subsequent Smad signaling pathways^27^,^28^. Indeed, BAMBI expression can be upregulated by TGF-β in a feedback loop^28^, thus making the prediction of the function or mode of this pseudo-receptor difficult. A potential involvement of BAMBI in inflammatory response has been proposed^29^, and its deficiency protects mice against the development of autoimmune arthritis by the modulation of Th17-Treg differentiation^30^. However, little is known regarding their role in human immunity and ultimately their capacity to influence the Th17-Treg axis and immune balance in COPD.

In light of our initial findings both *in vivo* and *in vitro*^14^,^15^,^31^,^32^, the present study was initiated to further understand the contribution of immune imbalance to COPD. Given its counteractive influence on TGF-β signaling, we hypothesized that BAMBI may play an important regulatory role in the internal environment of COPD. By assessing peripheral blood samples from donors with or without airway obstruction, we confirmed an immune imbalance of peripheral Th17/Tregs and TGF-β signaling in COPD. Our data further suggest that BAMBI is expressed in both peripheral CD4^+^ T cells and plasma and that upregulated BAMBI could be linked to the Th17/Treg balance through the TGF-β/BAMBI pathway in smokers who develop COPD.

## Methods

### Subjects

In total, 27 healthy asymptomatic non-smoking control subjects with normal lung function (HC), 24 asymptomatic smokers with normal lung function (HS) and 29 patients with stable COPD were recruited (Table 1). Depending on cell availability, mRNA profiles were studied in a subset of these patients (10 of 29, Table 2). COPD patients were diagnosed according to the criteria supplied by the Global Initiative for Chronic Obstructive Lung Disease (GOLD) guidelines^1^ and were free of exacerbation for at least 4 weeks before the study. The number of peripheral white blood cells (WBC, Table 1 and 2) and the frequency of neutrophils in WBC were obtained from routine blood examination by Laboratory Department of our hospital. Only the donors within normal WBC levels (4~10 × 10^9^/L) were recruited, in order to exclude the potential of infection and exacerbation. COPD patients and HS had a current smoking habit for 10 pack-years or more. Individuals with malignant tumors, type 1 diabetes mellitus, inflammatory bowel disease, rheumatoid arthritis, or other immune-related diseases were excluded. This study was conducted in accordance with the Declaration of Helsinki, and was approved by the Ethics Committee of Union Hospital, Tongji Medical College, Huazhong University of Science and Technology (# 2013/S048). In addition, written informed consent was obtained from each subject.

### Sample collection and processing

Peripheral blood samples from each subject were collected in heparin-treated tubes (BD Biosciences, San Diego, USA) and used for plasma selection and cell isolation. Peripheral blood mononuclear cells (PBMCs) were isolated using Lymphocyte Separation Medium (MP Biomedicals, Illkirch, France). CD4^+^ T cells were isolated from PBMCs by MACS based on negative selection using a CD4^+^ T cell isolation kit (Miltenyi Biotec, Bergisch Gladbach, Germany) according to the manufacturer’s instructions. CD4^+^ T cell purity was >97% as measured by flow cytometry.

### Flow cytometry

PBMC marker expression was determined by flow cytometry as described previously^15^ after surface or intracellular staining with anti-human-specific Abs that were conjugated to Alexa Fluor 660, FITC, PerCP-cy5.5, PE, or PE-Cy7. These Abs included anti-CD3, anti-CD8, anti-CD25, anti-Foxp3, and anti-IL-17A as well as isotype mAbs, which were purchased from BD Biosciences or eBioscience (San Diego, USA). Flow cytometry detection of BAMBI was not possible as no commercial monoclonal Abs are currently available that specifically recognize human BAMBI. Intracellular staining was performed on PBMCs that had been stimulated with PMA (50 ng/ml; Sigma-Aldrich, St. Louis, USA) and ionomycin (1 μg/ml; Sigma-Aldrich) in the presence of GolgiStop (BD Biosciences) for 5 h. CD3^+^CD8^−^ T cells were treated as CD3^+^CD4^+^ T cells after preliminary experiments had shown that these two subsets are >95% congruent (data not shown). Flow cytometry was performed on a fluorescence-activated cell sorter (FACS) Canto II (BD Biosciences) and analyzed using BD FACSDiva Software and FCS Express 5 software (De Novo Software, Los Angeles, USA).

### Enzyme-linked immunosorbent assay (ELISA)

Plasma molecule concentrations were determined using sandwich ELISA kits according to the manufacturer’s protocols for active TGF-β (without acid treatment), total TGF-β (with acid treatment) (Boster, Wuhan China), TGF-β RI, TGF-β RII and BAMBI (Uscn Life Science, Wuhan, China). The limits of detection for TGF-β, TGF-β RI, TGF-β RII and BAMBI were 1 pg/ml, 0.121 ng/mL, 0.115 ng/mL, and 0.057 ng/mL, respectively.

### Quantitative real-time PCR (qRT-PCR)

Quantitative determination of gene expression was performed by qRT-PCR as described previously^15^. In brief, total RNA was extracted from CD4^+^ T cells using RNAiso plus (TaKaRa, Dalian, China) and reverse transcribed into cDNA using a PrimeScript^TM^ RT Reagent Kit (TaKaRa) according to the manufacturer’s protocol. Subsequently, PCR amplification was performed on a StepOnePlus Real-Time PCR System (Applied Biosystems, Foster City, USA) using SYBR^®^ Premix Ex Taq^TM^ (TaKaRa) and specific primers (Table 3). The relative expression levels of each gene were normalized to GADPH using the 2^−∆Ct^ method (∆Ct = Ct _target gene_ − Ct _GADPH_)^33^. The qRT-PCR data were analyzed using StepOne software v2.3 (Applied Biosystems).

### Confocal microscopy

Double immunofluorescence staining was performed on CD4^+^ T cells to visualize TGF-β RI/BAMBI and TGF-β RII/BAMBI colocalization. Purified CD4^+^ T cells were spread on glass slides coated with poly-L-lysine and 3-aminopropyl-triethoxysilane (APES) for adhesion. The slips with adherent cells were fixed in 4% paraformaldehyde for 15 min at room temperature. After the cells were washed three times with PBS, they were permeabilized with 0.3% Triton X-100 in PBS for 15 min. Non-specific binding sites were blocked with 5% donkey serum in PBS for 30 min at room temperature. Cells were incubated with rabbit polyclonal primary Abs that were targeted against human TGF-β RI (Abcam, Cambridge, UK) and human TGF-β RII (Santa Cruz Biotechnology, Santa Cruz, USA) and goat polyclonal Abs targeted against human BAMBI (R&D Systems, Minneapolis, USA) overnight at 4 °C. Negative controls using isotype match primary antibodies were established. Then, the cells were incubated with the appropriate secondary Abs including FITC-conjugated AffiniPure donkey anti-rabbit IgG and TRITC-conjugated AffiniPure donkey anti-goat IgG (both were purchased from Protein Tech Group, Chicago, USA) for 60 min at room temperature in the dark. Nuclei were counterstained blue with DAPI staining solution (Boster). Finally, the slides were viewed under an Olympus FV500 confocal laser scanning microscope (Olympus, Tokyo, Japan).

### Statistics

The results are expressed as the means ± SEM unless otherwise indicated. The nonparametric Kruskal-Wallis test followed by Dunn’s post hoc test was used to test multiple comparisons between groups. Correlations between variables were determined using the Spearman rank test. Data analysis was performed using GraphPad Prism 6 software (GraphPad Software, La Jolla, USA), and a value of P < 0.05 (2-sided) was considered statistically significant.

## Results

### Th17/Treg imbalance was observed in peripheral blood samples from COPD patients

**Figure 1:**
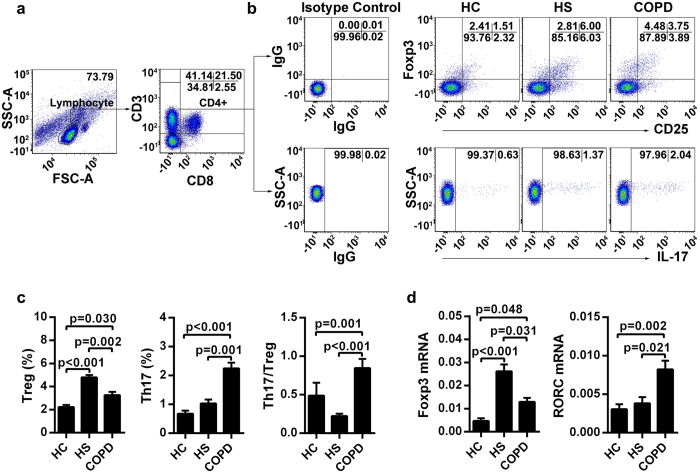
Imbalance of circulating Treg and Th17 cells in patients with COPD. (**a**) Lymphocytes were gated on FSC-A versus SSC-A plots, and CD4^+^ T cells were identified based on their expression of CD3 and not CD8. (**b**) Representative flow cytometric dot-plots of Treg and Th17 cells in peripheral blood. (**c**) Comparisons of Treg and Th17 cell percentages in peripheral blood from healthy controls (HC, n = 27), healthy smokers (HS, n = 24) and COPD patients (n = 29). (**d**) qRT-PCR analysis of Foxp3 and RORC gene expression in peripheral CD4^+^ T cells of HC subjects (n = 10), HS subjects (n = 10) and COPD (n = 10) patients. The data are represented as the mean ± SEM; a value of P < 0.05 was considered statistically significant.

As shown in Fig. 1b,c, the percentage of CD4^+^ Tregs coexpressing CD25 and Foxp3 significantly increased in peripheral blood from HS subjects compared with that from COPD patients and HC subjects. Furthermore, the frequency of Tregs was markedly higher in COPD compared with HC.

In addition, as indicated in Fig. 1b,c, a higher proportion of CD4^+^ T cells expressing IL-17 was observed in peripheral blood from COPD compared with HC and HS subjects. No significant differences in circulating Th17 frequencies were observed between HC and HS subjects, although a trend toward higher levels was detected in peripheral blood from HS subjects compared with that from HC subjects.

Because the Th17/Treg ratio may be an effective marker to evaluate disease severity in both animal models and human diseases^10^, we also investigated the immune changes involved in the Th17/Treg balance. We observed that the Th17/Treg ratios displayed significantly higher upregulation in COPD patients than did those in both HC and HS groups (Fig. 1b,c).

In parallel, transcription factors of Treg-specific Foxp3 and Th17-specific RORC in peripheral CD4^+^ T cells had the same trend of variation as the proportion of each subset in different groups (Fig. 1d). The mRNA transcript results further reinforce the hypothesis of Th17/Treg imbalance in COPD patients.

### Plasma BAMBI protein up-regulation was first discovered in COPD patients

Because a number of molecules such as TGF-β1 and its receptors TGF-β RI and TGF-β RII may be involved in the development of COPD, we quantified these molecules in the plasma samples from COPD patients and controls.

**Figure 2:**
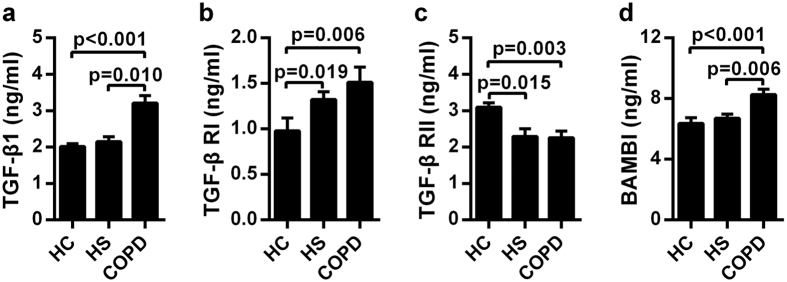
Disordered homeostasis of plasma TGF-β1 and its receptors in patients with COPD. Standardized sandwich ELISA of plasma from healthy controls (HC, n = 27), healthy smokers (HS, n = 24) and COPD patients (n = 29) were performed to assess the levels of (**a**) TGF-β1, (**b**) TGF-β1 RI, (**c**) TGF-β1 RII, and (**d**) BAMBI. The data are represented as the mean ± SEM; a value of P < 0.05 was considered statistically significant.

Quantification of the active form of TGF-β1 did not differ significantly in the three groups (HC: median = 0, range = 0–65.17; HS: median = 0, range = 0–66.28; COPD: median = 0, range = 0–87.66; pg/ml), demonstrating a low presence of these molecules in the entire population. Notably, as shown in Fig. 2a, total plasma TGF-β1 concentrations, which contained both the active and latent forms of TGF-β1, were statistically higher in COPD patients than in the other groups studied. These data indicate that the latent, but not the active, form of TGF-β1 is associated with COPD pathogenesis.

Because TGF-β signaling is activated by binding to TGF-β RII and TGF-β RI, we also evaluated the plasma levels of soluble receptors, which partially reflect the activated state of TGF-β signaling. We detected significantly increased plasma TGF-β RI concentrations in COPD patients and HS subjects compared with the HC group (Fig. 2b). In contrast, TGF-β RII plasma levels were decreased significantly in COPD and HS subjects compared with HC subjects (Fig. 2c). However, plasma TGF-β RI and RII levels in COPD patients were comparable with those of HS donors. The notable discrepancy of TGF-β receptor (RI and RII) levels illustrates that these two receptors are different in smokers and non-smoker, rather than in COPD and non-COPD.

As a TGF-β pseudo-receptor, BAMBI may have a regulatory or inhibitory effect on TGF-β proteins; thus, we investigated the role of plasma BAMBI protein in COPD (Fig. 2d). Interestingly, we were the first to observe that BAMBI expression was significantly higher in COPD patients compared with HS and HC subjects.

### Circulating CD4^+^ T cells in COPD patients displayed altered mRNA expression of TGF-β signaling molecules

**Figure 3:**
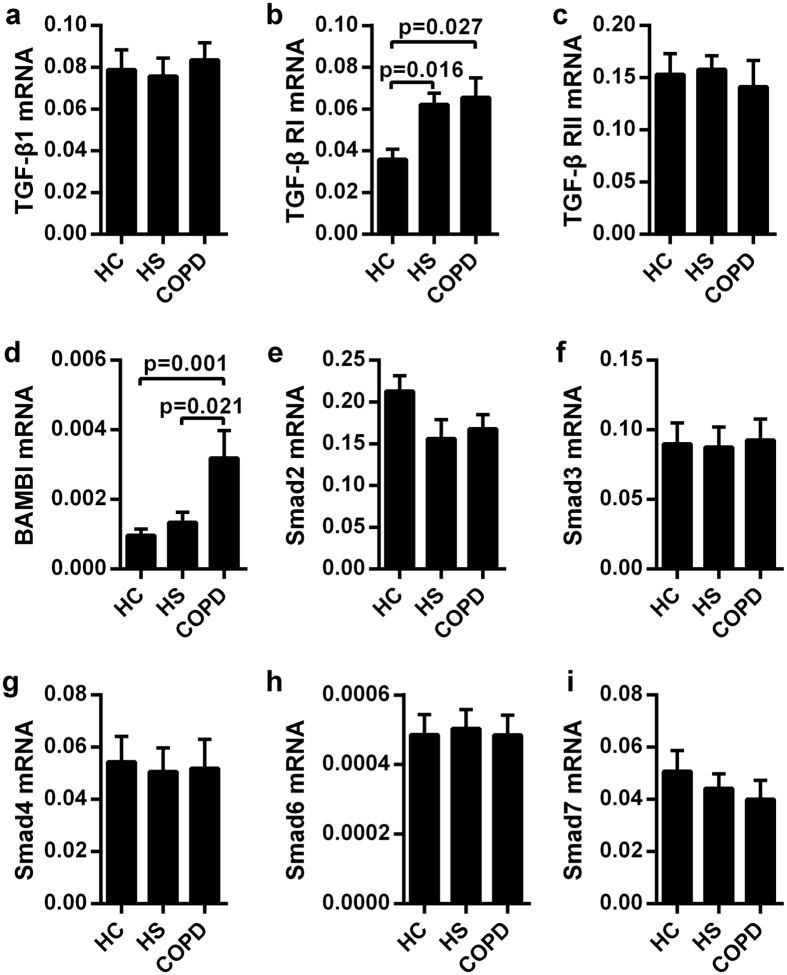
Altered mRNA expression of TGF-β signaling molecules by peripheral CD4^+^ T cells in patients with COPD. Quantitative real-time PCR analysis of circulating CD4^+^ T cells from healthy control subjects (HC, n = 10), healthy smokers (HS, n = 10) and COPD patients (n = 10) were performed to measure the mRNA levels of (**a**) TGF-β1, (**b**) TGF-β1 RI, (**c**) TGF-β1 RII, (**d**) BAMBI, (**e**) Smad 2, (**f**) Smad 3, (**g**) Smad 4, (**h**) Smad 6, and (**i**) Smad 7. The data are presented as the mean ± SEM; a value of P < 0.05 was considered statistically significant.

We investigated the role of TGF-β1 from peripheral blood CD4^+^ T cells in COPD. Quantitative evaluation of TGF-β1 mRNA levels normalized to GADPH transcripts demonstrated no significant differences among the three groups (Fig. 3a).

In contrast to TGF-β1, both COPD patients and HS individuals had significantly higher TGF-β RI expression than did HC subjects (Fig. 3b). In contrast, TGF-β RII mRNA levels were relatively low and similar among the three groups, suggesting a minor association between the mRNA level of this regulatory molecule and disease/smoking status (Fig. 3c).

Figure 3b,d showed that in healthy subjects, relative BAMBI mRNA expression was only approximately one-thirtieth of that of TGF-β RI (0.00097 ± 0.00018 vs. 0.036 ± 0.005, respectively; mean ± SEM). In particular, BAMBI mRNA levels in the peripheral CD4^+^ T cells from COPD were significantly increased compared with the other groups (Fig. 3d), which might link the imbalance between the pseudo-receptor and the progression of COPD.

We then studied TGF-β1 downstream signaling components, among which the Smads play a pivotal role. However, no significant changes in Smad mRNA levels were observed among the different groups (Fig. 3e–i).

### BAMBI protein was detectable and displayed a typical membrane-bound pattern in peripheral CD4^+^ T cells

**Figure 4:**
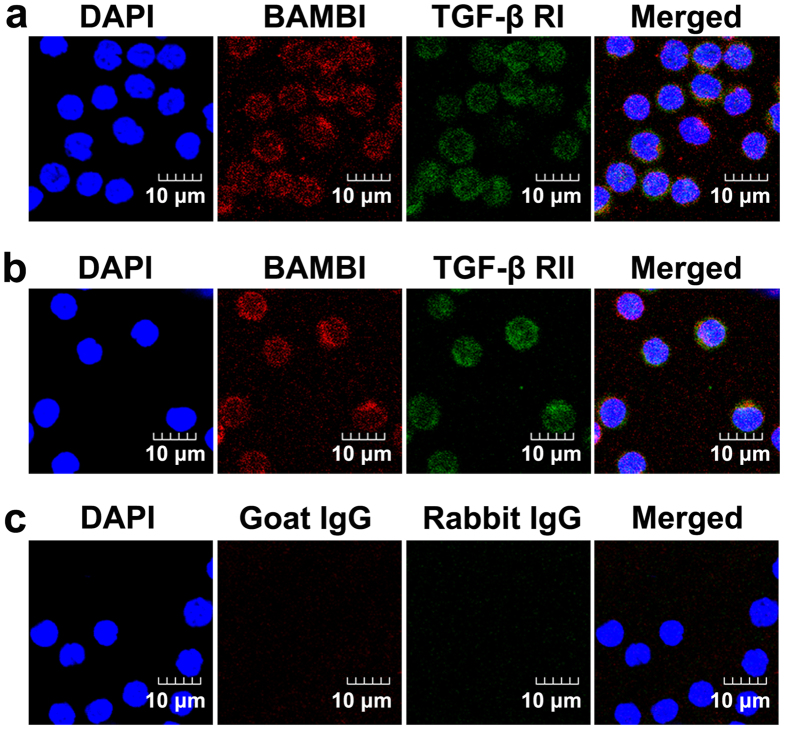
Immunofluorescence localization of BAMBI in peripheral CD4^+^ T cells. Isolated CD4^+^ T cells were incubated with goat polyclonal Ab targeted against BAMBI and then were stained with TRITC-conjugated AffiniPure donkey anti-goat IgG (red). Colocalization of TGF-β receptors was visualized by (**a**) rabbit anti-TGF-β RI or (**b**) anti-TGF-β RII polyclonal antibody and FITC-labeled donkey anti-rabbit IgG (Green). DAPI staining solution was used for cell nuclei staining. The specificity of the immunostainings was demonstrated using isotype match controls (**c**).

Confocal fluorescence microscopy revealed that all circulating CD4^+^ T cells were positive for TGF-β RI, TGF-β RII and BAMBI, which were significantly expressed at relatively high levels from COPD patients, HS subjects, and even in healthy controls (Fig. 4). Thus, a detailed quantification among the different groups could not be performed. Notably, BAMBI (red staining) was observed in a typical membrane-bound distribution with a punctate pattern and was colocalized with TGF-β RI and TGF-β RII (green staining). No immunostaining was detected with isotype controls (Fig. 4c).

### Plasma BAMBI levels in COPD patients correlated positively with plasma TGF-β1 levels and with the Th17/Treg ratio

**Figure 5:**
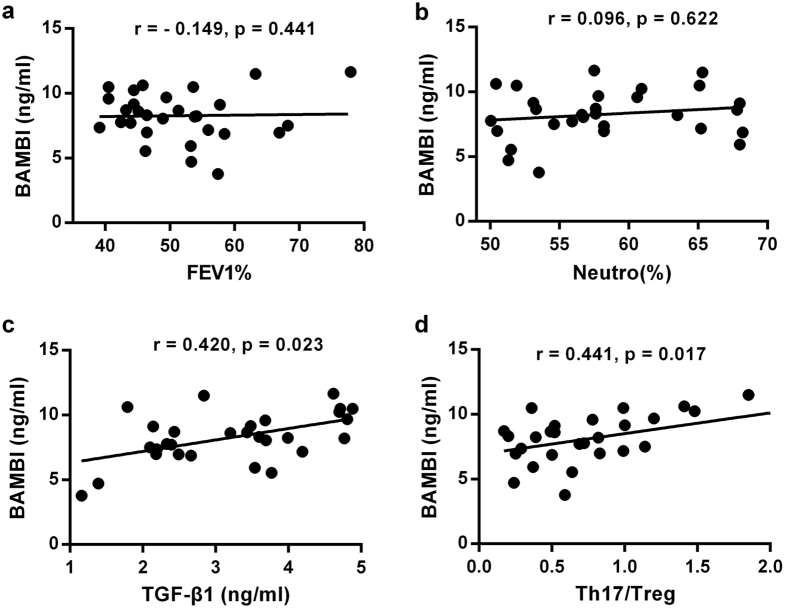
Correlation between plasma BAMBI levels and clinicopathological parameters of patients with COPD. Correlation of (**a**) FEV1% predicted value, (**b**) neutrophil percentage in peripheral white blood cells, (**c**) plasma TGF-β1 levels, and (**d**) Th17/Treg ratio with plasma BAMBI levels in COPD patients (n = 29). Each symbol represents one individual COPD patient (black dots); a value of P < 0.05 was considered statistically significant.

Single regression analysis between plasma BAMBI levels and clinicopathological parameters in COPD patients was performed. We observed that plasma TGF-β1 levels displayed a positive correlation with increased plasma BAMBI levels (Fig. 5c), supporting a possible role for TGF-β1 in the induction of TGF-β pseudo-receptor^28^. Interestingly, positive correlations were found between plasma BAMBI levels and the Th17/Treg ratio (Fig. 5d), which might reflect potential link between BAMBI and Th17/Treg imbalance. However, plasma BAMBI levels did not demonstrate any correlation with the percentage of neutrophils or the FEV1% predicted value (Fig. 5a,b), which suggested a lesser contribution of BAMBI to neutrophils inflammation or lung function loss.

## Discussion

Previous studies have demonstrated key roles for BAMBI in human diseases such as fibrosis^34^,^35^, adipogenesis^36^,^37^ and certain cancers^38^,^39^,^40^. Interestingly, our data are the first demonstration that BAMBI is expressed in both circulating CD4^+^ T cells and plasma and may be characterized as a novel immune regulator in the context of COPD. Strikingly, we reported here that BAMBI expression was significantly stronger in COPD patients and that increased plasma BAMBI levels in COPD patients displayed excellent correlations with enhanced plasma TGF-β1 levels and with the Th17/Treg ratio. These results suggest that impaired TGF-β signaling might induce the Th17/Treg imbalance in the peripheral blood, which might disturb immune homeostasis in smokers who develop COPD.

Tregs and their effector molecules such as TGF-β1 have been identified as vital immune modulators that efficiently maintain immune homeostasis, avoiding unnecessary reactions^41^,^42^. Consistent with our present study, increased Foxp3^+^ Tregs have been observed in the peripheral blood of COPD patients compared with HC subjects^13^,^43^,^44^. Interestingly, we even detected an increase in the circulating Treg population in HS subjects without airway obstruction compared with COPD. These results suggest that the immune system may attempt to attenuate smoke-induced inflammatory responses at the initiation phase via feedback regulation of Tregs. In contrast, this mechanism of peripheral tolerance becomes overwhelmed in susceptible individuals by the persistence of CS exposure, thus leading to an enhanced inflammatory response in COPD patients. However, several previous studies on the immune responsibility of Tregs demonstrated controversial results indicating significantly decreased levels of peripheral Tregs in COPD patients compared with healthy subjects^9^,^16^. Understandably, the lack of consensus may partly arise from different experimental models, a limited sample size and various methods for detecting Tregs.

Th17 cells are now widely accepted to be crucial for regulating diverse chronic immune diseases^45^. Interest in the therapeutic potential of Th17 cells in COPD has increased because Th17 cells exert direct influence on epithelial cells, smooth muscle cells and airway fibroblasts to induce neutrophil chemokine secretion^46^,^47^. However, when modulating Th17 cells activity, we should keep in mind that blockade of IL-17 could be associated with an increased risk of tumor activity as well as for opportunistic infections^48^. As reported previously^13^,^49^, our current study observed an increase in the proportion of circulating Th17 cells from COPD patients compared with those from HC and HS subjects. Meanwhile, a trend to an increase was observed in peripheral Th17 cells in HS compared with HC, although without a significant difference. These finding demonstrate Th17 polarization only in a susceptible minority of tobacco smokers. The antigenicity of foreign or self-molecules (e.g., elastin, collagen or endothelial fragments) could be enhanced in susceptible smokers, thereby activating more effector cells and breaking the lung tolerogenic state^50^. Moreover, the Th17/Treg ratio, a novel parameter that was reported to be inversely related to pulmonary function^9^, was markedly higher in COPD patients than in HC and HS subjects. We hypothesize that Th17/Treg imbalance may rely on intrinsic T cell factors or on the cytokine microenvironment during antigen presentation.

Although previously considered to promote the development of airflow limitation through fibrosis^51^, TGF-β1 has recently been considered a protective cytokine by switching off the inflammatory response^52^ and by downregulating mucin production^53^. In accordance with our current work^20^,^21^, significantly increased plasma total TGF-β1 levels were observed in COPD patients, together with an upregulated trend in HS subjects, reflecting a feedback regulation by which smoking-induced chronic inflammation promoted high TGF-β1 production levels. Additionally, a previous study demonstrated upregulated TGF-β receptor expression by peripheral T cells in COPD compared with HC^21^, although this study failed to note the receptor type. Similarly, compared with the HC group, COPD patients and HS donors had elevated TGF-β RI protein from plasma and increased TGF-β RI mRNA in CD4^+^ T cells. Conversely, TGF-β RII plasma levels were significantly lower in COPD patients and HS subjects than in HC subjects, comparable to the previous finding that CSE could downregulate TGF-β RII expression, thus blocking cellular responsiveness to TGF-β1^54^. Nevertheless, notably, no significant differences in TGF-β RI/RII expression levels were detected between COPD patients and HS donors. Therefore, the discrepancy in TGF-β RI/RII levels may be related to smoking status (smokers vs. non-smoker) rather than to disease status (COPD vs. non-COPD).

At the molecular level, BAMBI negatively influences the TGF-β/smad signaling pathway system^27^,^28^. Of particular interest, BAMBI mRNA instability may relate to AU-rich sequences (ARE)^29^, which were recently proposed to regulate the half-life of proinflammatory genes^55^. A latest report identifies BAMBI as a TGFβ rheostat that regulates Th17/Treg differentiation and the development of autoimmune arthritis by weakening IL-2 signaling^30^. Meanwhile, COPD lungs have strong BAMBI expression, which is upregulated after *in vitro* infection^26^. Our present study is the first demonstration of BAMBI expression in both circulating CD4^+^ T cells and plasma, with BAMBI protein expression demonstrating a typical membrane-bound, punctate pattern and colocalization with TGF-β RI and TGF-β RII. Strikingly, peripheral blood from COPD patients displayed significantly stronger BAMBI, but not TGF-β RI/RII, expression compared with that from smokers without airway obstruction and from healthy donors. Perhaps the combination of cigarette-mediated proinflammation and TGF-β inhibition by BAMBI binding may influence inflammation. Thus, given the roles of TGF-β in Th17/Treg differentiation and the inflammatory response^19^ together with the altered BAMBI expression, the involvement of the TGF-β/BAMBI pathway in COPD inflammation is a reasonable possibility. Notably, the mRNA expression of downstream Smads in CD4^+^ T cells did not show significant changes among the groups, which we presume primarily reflects differences in phosphorylation levels without necessarily directly altering total Smad gene expression.

Importantly, we assessed the regulatory functions of plasma BAMBI levels within COPD patients in relation to clinicopathological parameters. The plasma BAMBI levels did not correlate with the neutrophil proportion or FEV1% predicted value, which might reflect a lesser link between BAMBI imbalance and neutrophilic inflammation or lung function. However, the lack of correlation between plasma BAMBI and neutrophils frequency may be due to the limited volunteers within normal ranges of WBC levels. Interestingly, the levels of plasma BAMBI displayed excellent positive correlations with elevated plasma TGF-β1 levels and with the Th17/Treg ratio. Herein, we linked the imbalance between altered TGF-β signaling via BAMBI and the differentiation of Th17/Treg. TGF-β1 likely upregulates BAMBI expression^28^, and upregulated BAMBI may relatively weaken TGF-β signaling as a negative feedback loop^27^,^28^. Therefore, blunted TGF-β signaling in Th cells might result in a biased Th17/Treg balance in COPD patients. Hypothetically, Tregs and Th17 effectors arise in a mutually exclusive fashion depending on the microenvironment *in vivo*. In the absence of any inflammatory insult or at the steady-state level, TGF-β produced in the immune system will suppress effector T cell generation and induce protective Tregs to maintain homeostasis. However, upon inflammation or cigarette smoke exposure, BAMBI expression becomes upregulated by the activated immune system, which will suppress TGF-β-induced Treg generation and induce a pathogenic T cell response predominated by Th17 cells in susceptible individuals.

## Conclusions

Taken together, our data suggest that COPD presents not only a functional imbalance between Th17 and Tregs but also a TGF-β-BAMBI signaling pathway disorder, which is somewhat surprising. Notably, one may speculate that the TGF-β-BAMBI pathway plays an important role in a biased Th17/Treg balance away from a regulatory toward an inflammatory phenotype. These results offer an alternative explanation for the substantial activation of immune cells in COPD. Therefore, further mechanistic investigations, including gene knockdown /overexpression of human T cells *in vitro* as well as transgenic mice *in vivo*, are warranted to clarify the mechanism by which BAMBI exerts its effects.

## Additional Information

**How to cite this article**: Zhang, J.-C. *et al*. TGF-β/BAMBI pathway dysfunction contributes to peripheral Th17/Treg imbalance in chronic obstructive pulmonary disease. *Sci. Rep.* **6**, 31911; doi: 10.1038/srep31911 (2016).

## Figures and Tables

**Table 1:** Demographics and clinical characteristics of all participants.

Variables	HC	HS	COPD
Subjects (No.)	27	24	29
Age (year)	56.5 ± 1.6	59.1 ± 0.9	58.9 ± 1.3
Gender (Male/female)	24/3	22/2	25/4
Tobacco (pack-year)	—	38 (10–68)	41 (12–80)
FEV1 (% predicted)	94.4 ± 0.6	94.3 ± 0.6	51.4 ± 1.7*^#^
FEV1/ FVC (%)	84.29 ± 1.40	80.88 ± 1.25	43.37 ± 2.04*^#^
WBC (× 10^9^/L)	6.66 ± 0.26	5.87 ± 0.23	6.03 ± 0.27

The data are represented as the mean ± SEM or median (range). FEV1: forced expiratory volume in one second; FVC: forced vital capacity; WBC: white blood cell. *P < 0.01 vs. the HC group; ^#^P < 0.01 vs. the HS group.

**Table 2:** Characteristics of participants used for mRNA profile analysis.

Variables	HC	HS	COPD
Subjects (No.)	10	10	10
Age (year)	60.0 ± 1.5	57.2 ± 1.1	59.1 ± 1.6
Gender (Male/female)	9/1	9/1	8/2
Tobacco (pack-year)	—	39 (17–53)	39 (23–68)
FEV1 (% predicted)	93.9 ± 1.2	92.3 ± 1.1	52.1 ± 1.9*^#^
FEV1/ FVC (%)	85.90 ± 2.43	84.93 ± 1.90	43.82 ± 3.46*^#^
WBC (×10^9^/L)	5.88 ± 0.35	6.12 ± 0.43	6.44 ± 0.51

The data are represented as the mean ± SEM or median (range). FEV1: forced expiratory volume in one second; FVC: forced vital capacity; WBC: white blood cell. *P < 0.01 vs. the HC group; ^#^P < 0.01 vs. the HS group.

**Table 3:** Primer sequences and product sizes of real-time PCR.

Gene name	Primer sequence (5′–3′)	Product length (bp)
Foxp3	F: CTGGCAAATGGTGTCTGCAAGT	107
	R: CTGCCCTTCTCATCCAGAAGATG	
RORC	F: CTGCAAGACTCATCGCCAAAG	83
	R: TTTCCACATGCTGGCTACACA	
TGF-β1	F: GCGACTCGCCAGAGTGGTTA	143
	R: GTTGATGTCCACTTGCAGTGTGTTA	
TGF-β RI	F: GGACCCACTTCCATTTCCTTC	125
	R: CCATCCCACTCCTCATCCA	
TGF-β RII	F: GAAATTCCCAGCTTCTGGCTCA	143
	R: CTGTCCAGATGCTCCAGCTCAC	
BAMBI	F: CATACCCACATTGGAATGCTGTC	144
	R: TGCACCTTGGTGATAAGGTTTCTG	
Smad2	F: GGCTTTACAGACCCATCAAATTCA	161
	R: GCACTATCACTTAGGCACTCAGCA	
Smad3	F: CCAGGGCTTTGAGGCTGTCTA	143
	R: GCAAAGGCCCATTCAGGTG	
Smad4	F: CAGCTATGCCAGAAGCCAGA	81
	R:GAACTCCTGGGACTTTCAACTGAC	
Smad6	F: GGACAAAACAAGAAAGACGCACT	83
	R: AAGGAAGAGGAAGGAGGAAGAGAA	
Smad7	F: CTGTCCAGATGCTGTGCCTTC	126
	R: TATGCCACCACGCACCAGT	
GAPDH	F: GCACCGTCAAGGCTGAGAAC	138
	R: TGGTGAAGACGCCAGTGGA	

All of the primers were synthesized by TaKaRa Biotechnology (Dalian).
